# Detection of somatic mutations in cell-free DNA in plasma and correlation with overall survival in patients with solid tumors

**DOI:** 10.18632/oncotarget.21982

**Published:** 2017-10-24

**Authors:** Meenakshi Mehrotra, Rajesh R. Singh, Sanam Loghavi, Dzifa Yawa Duose, Bedia A. Barkoh, Carmen Behrens, Keyur P. Patel, Mark J. Routbort, Scott Kopetz, Russell R. Broaddus, L. Jeffrey Medeiros, Ignacio I. Wistuba, Rajyalakshmi Luthra

**Affiliations:** ^1^ Department of Hematopathology, The University of Texas MD Anderson Cancer Center, Houston, Texas, USA; ^2^ Department of Translational Molecular Pathology, The University of Texas MD Anderson Cancer Center, Houston, Texas, USA; ^3^ Department of Thoracic/Head and Neck Medical Oncology, The University of Texas MD Anderson Cancer Center, Houston, Texas, USA; ^4^ Department of Gastrointestinal Medical Oncology, The University of Texas MD Anderson Cancer Center, Houston, Texas, USA; ^5^ Department of Pathology, The University of Texas MD Anderson Cancer Center, Houston, Texas, USA

**Keywords:** cfDNA, next-generation sequencing, genotyping, ddPCR, MassARRAY

## Abstract

A suitable clinical-grade platform is required for detection of somatic mutations with high sensitivity in cell-free DNA (cfDNA) of patients with solid tumors. In this study, we evaluated in parallel ultra-deep NGS with MassARRAY and allele-specific droplet digital PCR (ddPCR) for cfDNA genotyping and correlated cfDNA yield and mutation status with overall survival (OS) of patients. We assessed plasma samples from 46 patients with various advanced metastatic solid tumors and known mutations by deep sequencing using an Ampliseq cancer hotspot panel V2 on Ion Proton. A subset of these samples with DNA availability was tested by ddPCR and UltraSEEK MassARRAY for mutation detection in 5 genes (IDH1, PIK3CA, KRAS, BRAF, and NRAS). Sixty one of 104 expected tissue mutations and 6 additional mutations not present in the tissue were detected in cfDNA. ddPCR and MassARRAY showed 83% and 77% concordance with NGS for mutation detection with 100% and 79% sensitivity, respectively. The median OS of patients with lower cfDNA yield (74 vs 50 months; *P* < 0.03) and cfDNA negative for mutations (74.2 vs 53 months; *p* < 0.04) was significantly longer than in patients with higher cfDNA yield and positive for mutations. A limit-of-detection of 0.1% was demonstrated for ddPCR and MassARRAY platforms using a serially diluted positive cfDNA sample. The MassARRAY and ddPCR systems enable fast and cost-effective genotyping for a targeted set of mutations and can be used for single gene testing to guide response to chemotherapy or for orthogonal validation of NGS results.

## INTRODUCTION

Cancer is a microcosm of evolution in which gene mutations that give a cell a growth advantage are selected and sequential alterations of genes eventually result in a cancer phenotype. Targeting genomic alterations as they evolve using novel therapeutic agents in a timely manner is a key for successful precision cancer medicine [1–3]. Tumor biopsy specimens are currently considered to be the gold standard for pathological diagnosis as well as tumor genotyping for analysis of diagnostic, predictive and prognostic biomarkers. Tissue biopsy, however, is invasive and is associated with a risk of complications due to the biopsy procedure itself. At the cellular level, there are also challenges with excisional biopsy specimens such as intra-tumor heterogeneity and discrepancies in the genetic profiles between the primary neoplasm and its metastases [3].

Circulating cell-free DNA (cfDNA) analysis has emerged recently as a potential noninvasive alternative to tissue biopsy for tumor genotyping in patients with advanced solid tumors, due to ease of sample collection, availability, and shorter turnaround time [4–8]. Plasma cfDNA is secreted into the circulation by tumor cells and cells in the tumor microenvironment that undergo apoptosis or necrosis. Levels of circulating tumor DNA (ctDNA) have been shown to correlate with the degree of tumor burden and may contain DNA mutations derived from both primary and metastatic [9–11]. However, there is still limited data on the comparison of paired tumor and plasma samples that is needed to establish the clinical validity for cfDNA-based genotyping [12, 13]. Factors that may affect concordance between tissue biopsy specimens and cfDNA for a given somatic mutation include limited assay sensitivity, limited sample volume, a low overall contribution of DNA from the tumor into plasma, intra-tumor heterogeneity, subclonal mutation, tumor and plasma samples being obtained at different time points, and the potential evolution of a systemic tumor over time [1, 14].

Detection of mutations in ctDNA which can represent only a fraction of total cfDNA also imposes a challenge due to the signal to noise ratio and hence cfDNA genotyping requires highly sensitive platforms with a detection sensitivity of 0.1 to 0.01%. Next generation sequencing-based ultra-deep sequencing enables detection of a low amount of ctDNA in blood or other body fluids, but is restricted by errors introduced into the sample preparation and sequencing process [15], [16–19]. To overcome this challenge, various platforms have been developed. The MassARRAY (Sequenom, Inc), a medium-throughput multiplexed ultrasensitive mutation detection system based on MALDI-TOF, can be used to achieve a detection sensitivity of 0.1%. The process includes multiplex PCR, followed by mutation-specific, single-base extension. The captured and enriched products are then identified using matrix-assisted laser desorption/ionization time-of-flight mass spectrometry [20]. In contrast, the droplet digital PCR (ddPCR) platform, which uses a water–oil emulsion droplet system, offers easy workflow, better allele-specific sensitivity, and better precision and reproducibility than standard quantitative PCR [21]. Droplet digital PCR (ddPCR), is capable of detecting low-level mutations using low nucleic acid inputs, and can perform absolute quantification of mutant gene copy number in the background of wildtype sequence using allele specific probes or primers. However, ddPCR is limited in its multiplexing capability.

In this study, we detected mutations in plasma cfDNA by deep sequencing using the semiconductor-based Ion Proton NGS platform using cancer hotspot panel v2 and we compared the results with those of the UltraSEEK MassARRAY and ddPCR platforms for mutation detection sensitivity and specificity. We also compared the potential effects of cfDNA yield and cfDNA mutation status on overall patient survival.

## RESULTS

### Patient population clinical characteristics and cfDNA yield

The median age of the patients was 56 years (range, 25–82), and 27 (57%) were women. At last follow up, 25 (54%) patients were alive and 21 (46%) had deceased. The median time from tumor biopsy to plasma collection was 231.7 days (range, 0–3010 days). Tumor samples were obtained by resection (52%), biopsy (46%), or fine-needle aspiration (2%) from a variety of anatomic sites: lymph nodes (22%), lungs (17%), liver (15%) brain (13%), colon (9%), head and neck (7%), rectum (4%), breast (4%), pancreas (2%), femoral bone (2%), esophagus (2%), and spine (2%). These samples were derived from 19 (41%) primary, 19 (41%) metastatic, and 8 (17%) recurrent tumors. 22/36 (48%) cases showed metastasis to lymph nodes at time of primary diagnosis. 36 (78%) patients received surgery as primary therapy. 38 (82%) patients had undergone various modalities of treatment before tumor specimens were obtained, and 4 (9%) were on a single line of therapy. For 4 (9%) patients therapy data are not available (Table 1; Supplementary Table 1).

**Table 1:** Clinical characteristics of studied cohort (*N* = 46)

Characteristics	Number (%)
**Sex**	
Male	19 (41)
Female	27 (59)
Age at diagnosis, median, years	56 (range, 25–82)
**Specimen type**	
Resection	24 (52)
Biopsy	21 (46)
Fine-needle aspiration	1(2.1)
**Type of cancer**	
Brain	4
Breast	8
Colon	12
Head and Neck	4
Melanoma	8
Sarcoma	5
Others	1
**Primary vs. metastatic disease**	
Primary	19 (41)
Metastatic	19 (41)
Recurrent	8(17)
**Stage at tissue collection**	
1	2
2	5
3	5
4	18
Unknown	16
**Treatment modalities**	
Single line of therapy	4
Multiple line of therapy	38
Unknown	4
**Survival Status**	
Alive	25 (54)
Dead	21 (46)

**Figure 1:**
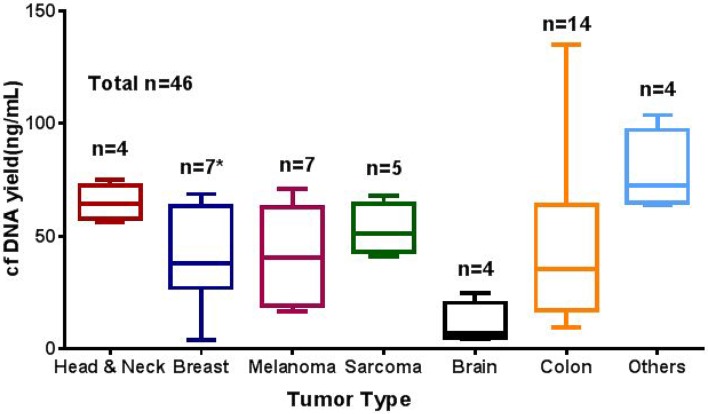
Average cfDNA yield (ng/mL) in different solid tumor types studied cfDNA extracted using the QIAamp Circulating Nucleic Acid Kit and quantified using the Qubit dsDNA HS Assay cfDNA yield (ng/mL). n represent the number tested for each tumor type. ^*^ one sample excluded from graph due to high cfDNA concentration (769 ng/mL).

cfDNA yield varied among different tumor types. The median amount of cfDNA isolated per mL of plasma was 39 ng per mL (range, 4–763.9 ng). Patients with brain tumors has lower yield as compared to other tumor type tested (Figure 1; Supplementary Table 1).

### Limit-of-detection studies for ddPCR and MassARRAY

**Figure 2:**
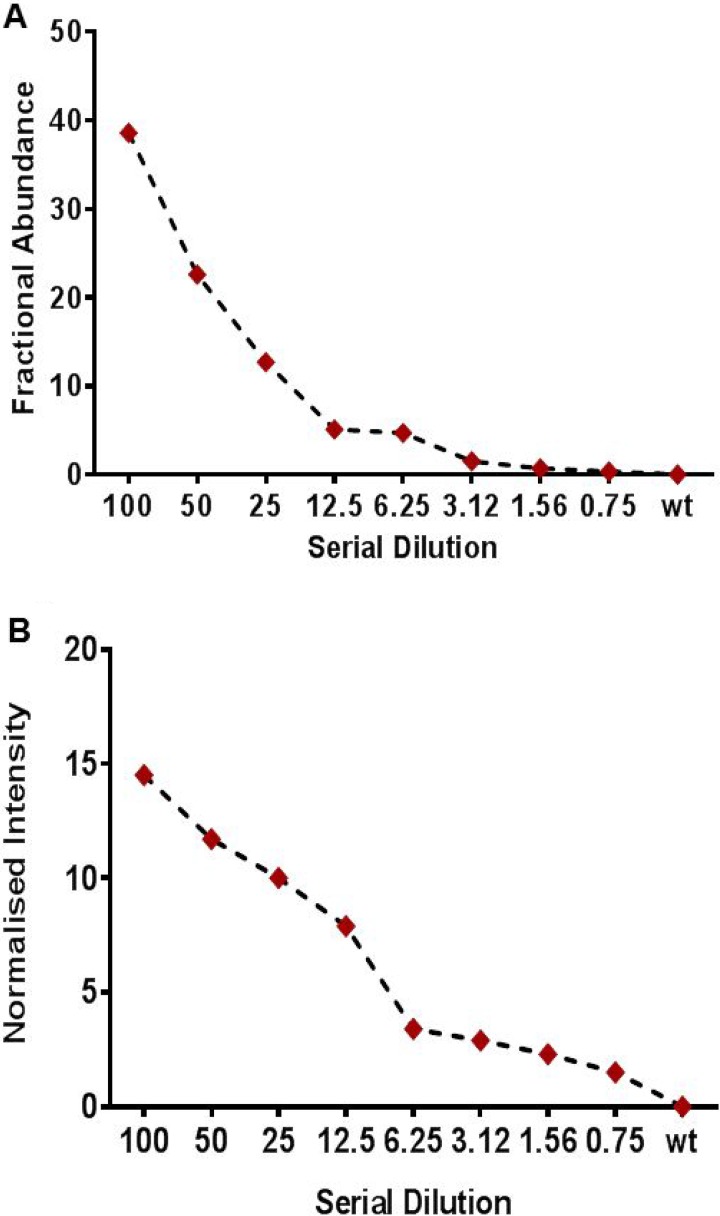
Limit of detection study (LOD) using a serially diluted patient positive sample for (**A**) Droplet digital PCR (ddPCR) (**B**) Sequenom based UltraSEEK MassARRAY assay for mutation detection in cfDNA samples. Patient cfDNA sample positive for *KRAS p.G12D* (33.7%) mutation was serially diluted to wild type cfDNA sample for LOD study. The positive cfDNA was diluted with a cfDNA sample negative for these mutations to obtain 50%, 25%, 12.5%, 6.25%, 3.15%, 1.5%, or 0.75% percent positive (mutated) DNA in wild-type DNA. For ddPCR allelic fraction was measured as Fractional abundance (FA) whereas for MassARRAY it was measured as Normalized intensity (NI).

To assess mutation detection sensitivity, we serially diluted a cfDNA sample positive for *KRAS* p.G12D (36%) into a cfDNA sample negative for this mutations to achieve dilutions of 50% (1:1 dilution), 25% (1:4), 12.5% (1:8), 6.25% (1:16), 3.12% (1:32), 1.5% (1:67), and 0.625% (1:160). These diluted samples were run on ddPCR and MassARRAY for detection of analytical sensitivity. Both the MassARRAY and ddPCR platforms detected the *KRAS* mutation at all dilutions tested and consistently showed a limit-of-detection of 0.1% variant allelic frequency in the background of wildtype (Figure 2A and 2B; Supplementary Table 2).

### Concordance between tumor and plasma DNA for detection of mutations

**Figure 3:**
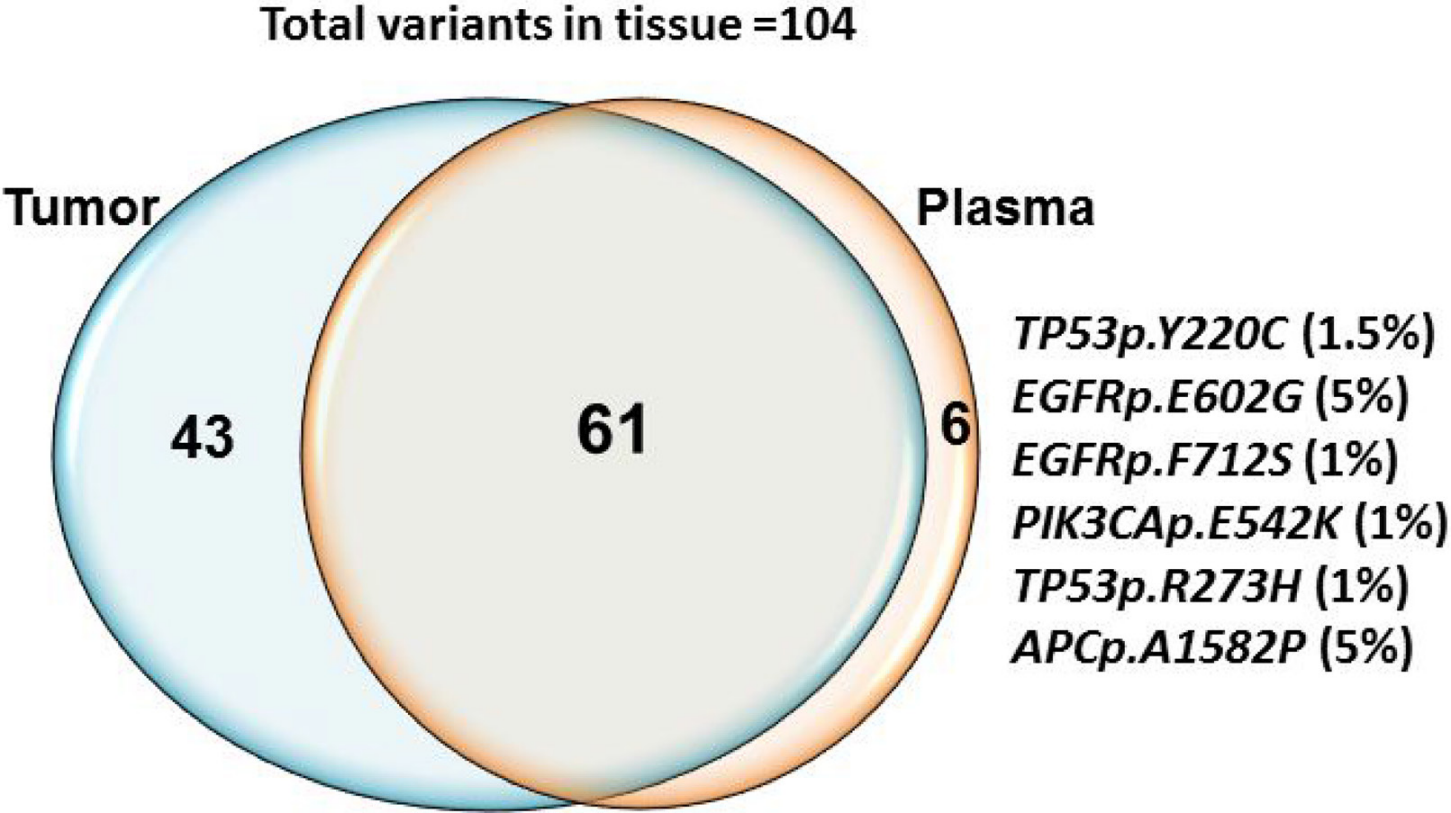
Concordance between mutations detected in tissue and plasma cfDNA by NGS Sixty one out of 104 expected mutations were detected in both tissue derived genomic DNA and plasma cfDNA. 43 mutations detected in tissue was not present in plasma. Six additional mutations *TP53p.Y220C* (1.5%) *EGFRp.E602G* (5%), *EGFRp.F712S* (1%), *PIK3CAp.E542K* (1.1%), *TP53p.R273H* (1%) and APCdupAp.I1311fs^*^4 (1.5%) were detected in plasma cfDNA only.

One hundred and four mutations in a number of genes were identified in tumor tissue using the Ion PGM platform and the Ion Ampliseq Cancer Hotspot Panel V2 (Supplementary Table 3). The median number of altered mutations per tumor was 2 (range, 1–4) and the median allelic fraction was 31% (range, 1.1–.84.3%). The most frequently mutated genes detected in tumor tissue in this cohort were *TP53* (26%)*, KRAS* (13%)*, PIK3CA* (13%), *APC* (10%) and BRAF (7%). Deep sequencing of 46 plasma samples using the semiconductor sequencing-based Ion Proton achieved an average output of 78.1M total reads. The mean sequencing depth across the samples was 8,860X. We detected 61 (59%) of 104 expected mutations and 6 additional mutations in plasma cfDNA which were not present in the tumor tissue specimen (Figure 3). *TP53* (16%)*, KRAS* (10%)*, PIK3CA* (11%), APC (5%) and BRAF (4%) were the most frequently mutated genes in this cohort in plasma (Supplementary Table 3). 32 (70%) plasma samples assessed showed a mutation in at least one of the gene mutations detected in the tissue biopsy specimen. The median number of altered mutations per plasma sample was 2 (range, 1–4), and the median detected VAF by NGS was 1.0% (range, 1–51.7%). In order to improve specificity and avoid false positive calls, we strictly set the cutoff to 1% allelic frequency, 250X coverage and 25 variant coverage for NGS and hence considered the calls below 1% as negative. Manual inspection in IGV was performed for each of the call. Fourteen of 46 patients showed discordance in mutation detection between the plasma cfDNA and tumor tissue. The explanation for this discordance may be related to the time lapse between obtaining the tissue biopsy specimen and plasma collection (range, 7 to 1,786 days) (Supplementary Table 4).

In patients in whom the tissue biopsy specimen was positive and cfDNA negative, manual inspection revealed few reads in some of discordant calls in plasma cfDNA (allelic fraction range 0.1 to 0.3%). We detected *TP53p.Y220C* (1.5%) *EGFRp.E602G* (5%), *EGFRp.F712S* (1%), *PIK3CAp.E542K* (1.1%), *TP53p.R273H* (1%) and *APCp.A1582P* (5%) mutations in cfDNA but not in tissue; in these cases no mutation reads were observed in the tissue-derived DNA by manual inspection in IGV. The samples in which additional clones detected were also positive for mutation in other expected variants. Time difference from tissue to plasma collection for the samples positive for additional clones in plasma ranges from 1 day to 127 days (Supplementary Table 4).

### Correlation between NGS, ddPCR and MassARRAY

**Figure 4:**
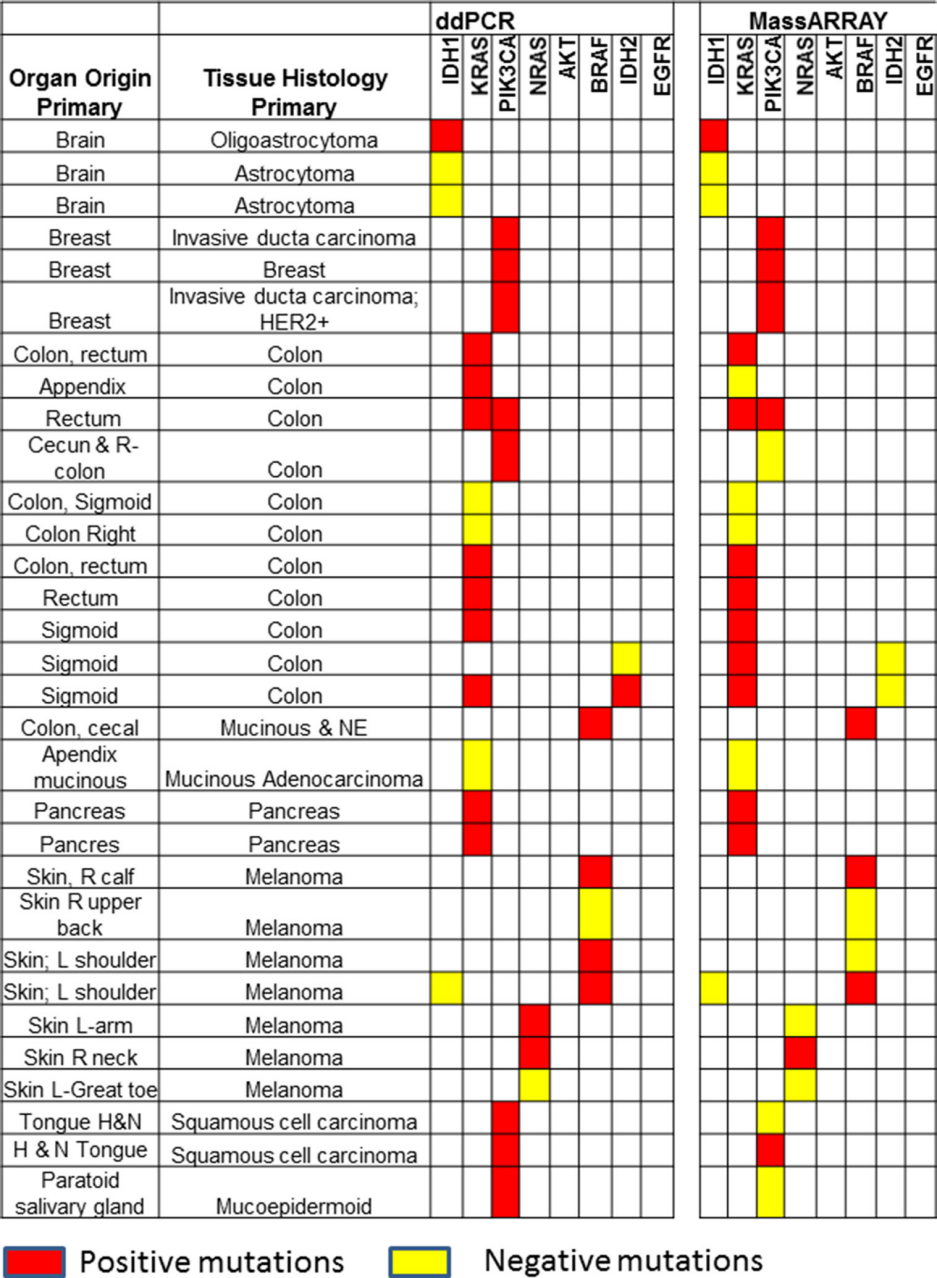
Comparison of ddPCR and ultraSEEK MassARRAY for mutation detection in plasma cell free DNA: A total of 35 variants in 31 samples were compared between ddPCR and MassARRAY MassARRAY showed 80% concordance for mutation detection with ddPCR (R^2^ = 0.9253; *p* < 0.0001). Red boxes represent positive mutations and yellow boxes represent negative mutations detected by ddPCR or MassARRAY.

**Figure 5:**
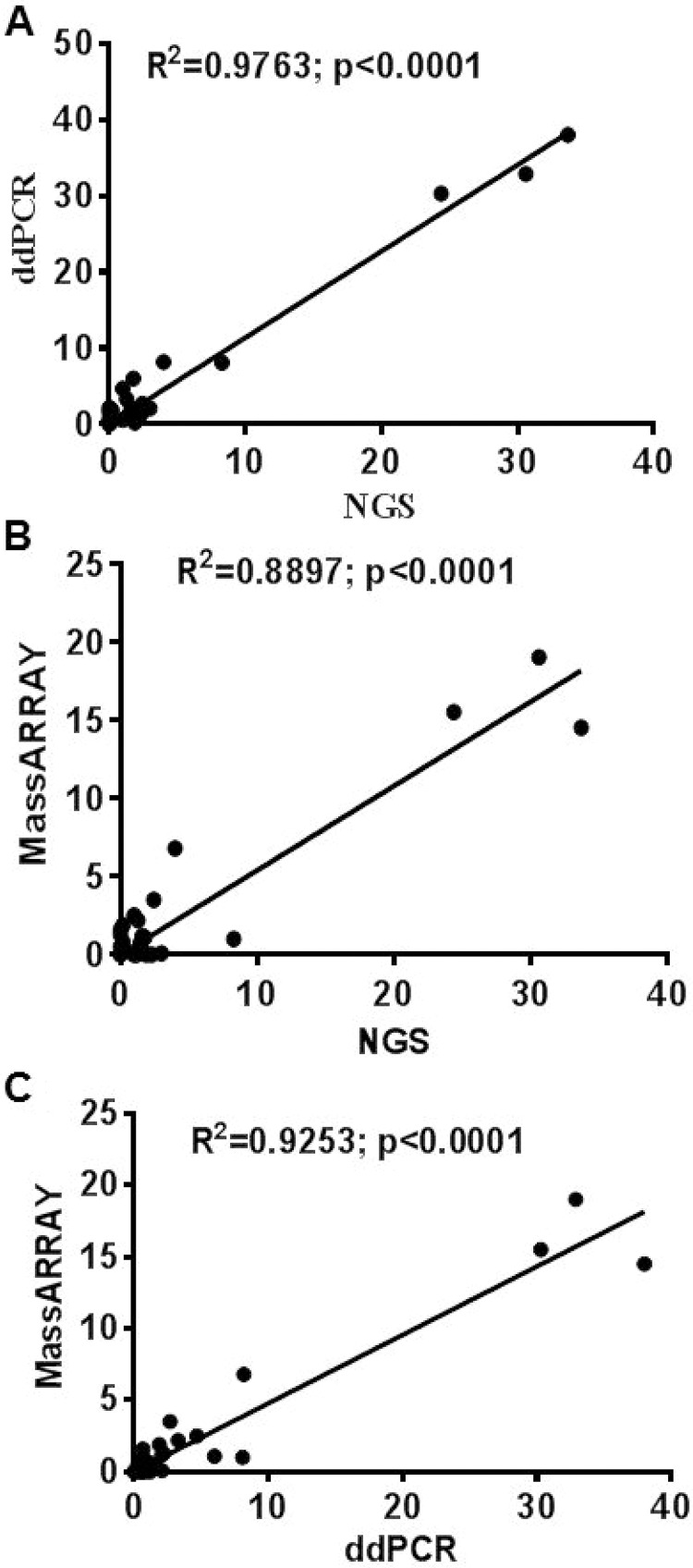
Correlation between ddPCR, UltraSEEK MassARRAY and NGS for mutation detection in plasma cfDNA Correlation established for 35 variants between NGS, ddPCR and MassARRAY. (**A**) ddPCR vs NGS (R^2^ = 0.9763; *p* < 0.0001) (**B**) UltraSEEK MassARRAY vs NGS (R^2^ = 0.8897; *p* < 0.0001) (**C**) UltraSEEK MassARRAY vs ddPCR (R^2^ = 0.9253; *p* < 0.0001).

Thirty-one cfDNA samples positive for *IDH1, KRAS, PIK3CA, NRAS, AKT, BRAF,* and *IDH2* mutations in tumor tissue were selected for ddPCR, MassARRAY and NGS comparison. A total of 35 variant calls, 19 (54%) positive and 16 (46%) negative for mutations detected by NGS in cfDNA samples were compared with ddPCR results. 25 calls were detected as positive and 10 negative for mutation by ddPCR. Due to higher analytical senstivity 6 variant calls (allelic fraction range 0.15–2.2%). which were not detected by NGS detected positive by ddPCR (Figure 4 and Supplementary Table 5). ddPCR showed 100% sensitivity, 63% specificity 76% positive predictive value and 100% negative predictive value for mutation detection as compared with the NGS platform for mutation detection (R^2^ = 0.9763; *p* < 0.0001) on plasma cfDNA (Figure 5A and Table 2).

**Table 2:** Clinical performance of ddPCR, MassArray and NGS test for mutation detection in plasma cfDNA

ddPCR vs NGS			
**ddPCR**	**NGS**		
		**Positive**	**Negative**
	**Positive**	19	6
	**Negative**	0	10
			
	**Statistic**	**Value**	**95% CI**
	**Sensitivity**	100.00%	82.35% to 100.00%
	**Specificity**	62.50%	35.43% to 84.80%
	**Positive Predicitive Value**	76.00% (^*^)	62.72% to 85.63%
	**Negative Predicitive Value**	100.00 % (^*^)	
			
		**MassARRAY vs NGS**	
**MassARRAY**	**NGS**		
		**Positive**	**Negative**
	**Positive**	15	4
	**Negative**	4	12

	**statistics**	**Value**	**95% CI**
	**Sensitivity**	78.95%	54.43% to 93.95%
	**Specificity**	75.00%	47.62% to 92.73%
	**Positive Predicitive Value**	78.95% (^*^)	60.87% to 90.04%
	**Negative Predicitive Value**	75.00 % (^*^)	54.56% to 88.23%
			
			
		**MassARRAY vs ddPCR**	
**MassARRAY**	**ddPCR**		
		**Positive**	**Negative**
	**Positive**	18	0
	**Negative**	7	10
			
	**Statistic**	**Value**	**95% CI**
	**Sensitivity**	72.00%	50.61% to 87.93%
	**Specificity**	100%	69.15% to 100.00%
	**Positive Predicitive Value**	100.00% (^*^)	53.70% to 85.36%
	**Negative Predicitive Value**	58.82 % (^*^)	43.24% to 72.82%

For MassARRAY, 35 variants detected by NGS were compared. 19 (54%) calls were detected as positive and 16 (46%) negative for mutation by MassARRAY (Supplementary Table 5 and Table 2). MassARRAY showed 79%%, sensitivity, 75% specificity, 79% positive predictive value, and 75% negative predictive value for mutation detection (R^2^ = 0.8897; *p* < 0.0001) as compared to NGS. (Figure 5B and Table 2). MassARRAY showed 80% concordance for mutation detection with ddPCR. MassARRAY confirmed 4 out of 6 new variant calls detected by ddPCR and missed 4 calls detected by NGS. MassARRAY showed 72% sensitivity, 100% specificity, 100% positive predictive value and 59% negative predictive value compared with ddPCR for cfDNA mutation detection (R^2^ = 0.9253; *p* < 0.0001) (Figure 5C, Table 2 and Supplementary Table 5).

### Correlation of cfDNA yield and mutation status to overall survival

**Figure 6:**
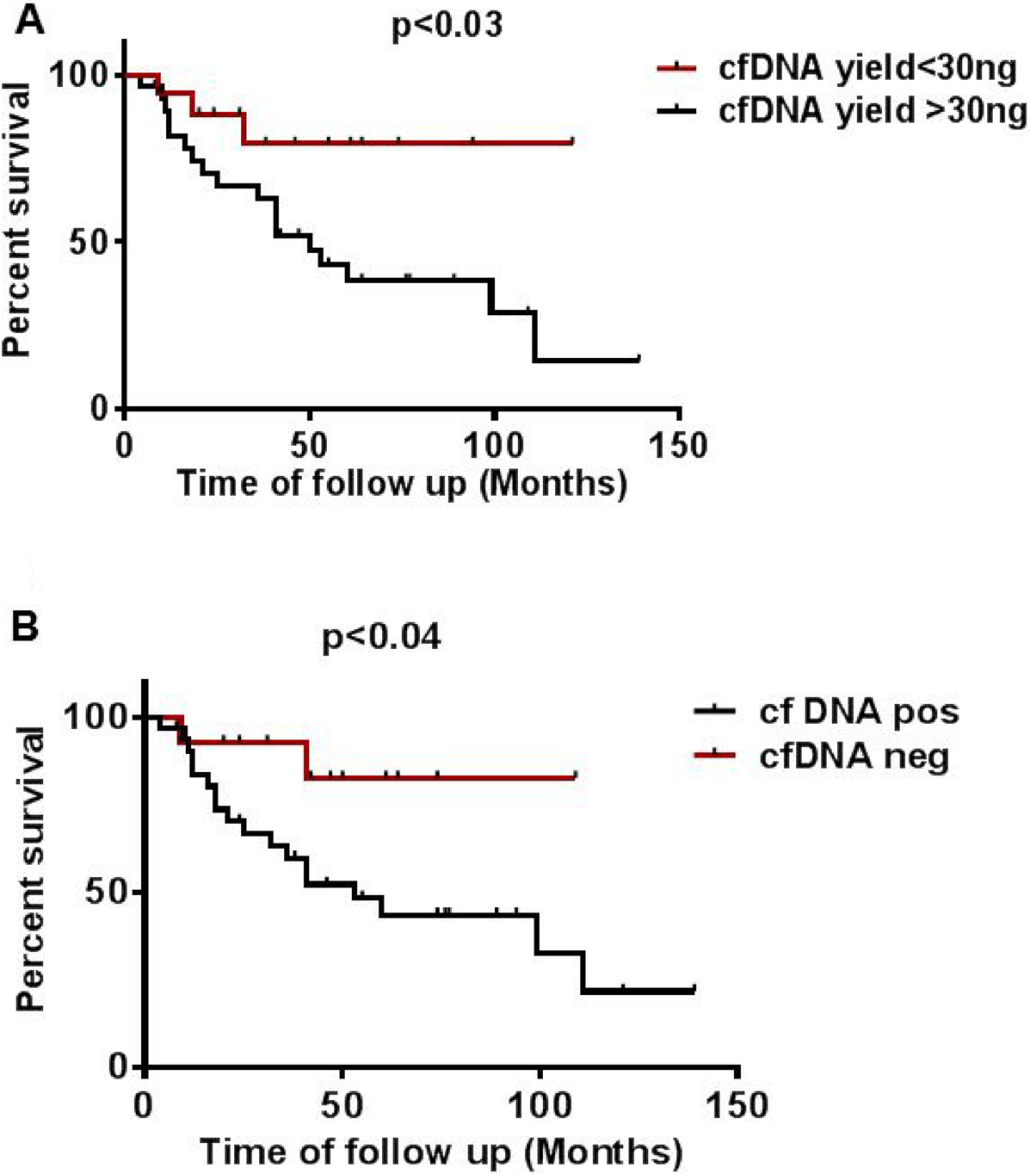
Effect of cfDNA yield and mutation status on overall survival (**A**) Effect of higher (> 30 ng) and lower (< 30 ng) cfDNA yield on overall survival (**B**) Effect of mutation status detected in cfDNA on overall survival calculated from time of collection of plasma in patients undertaken for study.

The amount of total cfDNA (ng/mL) and mutation status detected in cf plasma was associated with overall patient survival. We categorized samples into two subgroups: patients with < 30 ng and patients with > 30 ng cfDNA total yield per mL. The median OS of patients with < 30 ng was 74 months which was significantly longer than that of patients with > 30 ng which was 50 months. A multivariate analysis for all 46 patients showed a hazard ratio (HR) of 3.5 (95% CI 1.0 to 6.5) for samples with > 30 ng cfDNA yield as compared to a HR of 0.28 (95% CI 0.15 to 0.91; *p* < 0.03) for cfDNA yield < 30 ng (Figure 6A, Supplementary Table 1). Similarly, when we correlated cfDNA mutation status with OS we found that patients negative for mutation in cfDNA showed significantly a higher OS of 74.2 months as compared to patients positive for mutation in cfDNA that showed an OS of 53 months. A multivariate analysis for all 46 patients showed a HR of 3.88 (95% CI 1.0650 to 7.096) for cfDNA mutation positive samples as compared to a HR of 0.26 (95% CI 0.1409 to 0.9520, *p* < 0.04) for cfDNA negative samples (Figure 6B, Supplementary Table 1).

## DISCUSSION

Advancement in genotyping technologies and ultrasensitive detection methods has created great interest in the detection of somatic mutations in DNA from plasma, so-called “liquid biopsy”, for individualized patient management [3, 7]. Detection of genetic alterations associated with cancer in cfDNA and its concordance with mutations detected in tumor tissue biopsy specimens in several studies have suggested analysis of cfDNA for potential cancer biomarker in patients with solid tumors [22–26]. However, mutational analysis in cfDNA can be affected by many technical factors, such as low amount of cfDNA, lack of standardization of pre-analytic and analytical variables for implementation of cfDNA genotyping in the clinic, and background noise affecting the reliability of detecting low-level mutations [27, 28]. In the current study, we assessed plasma cfDNA for mutations in patients with advanced cancers with known tumor tissue specimen mutation status by deep sequencing using the Ion Proton platform. These plasma samples were subsequently used to test the ability and sensitivity of ddPCR and UltraSEEK MassARRAY platforms for mutation detection. We also compared the effect of cfDNA yield obtained in different tumor types tested and cfDNA mutation status on the overall survival of this patient cohort.

The most commonly altered mutations detected in plasma cfDNA were: TP53 (16%), *KRAS* (10%), *PIK3CA* (11%), *APC* (5%) and BRAF (4%), as would be as expected from the tumor types included in this study. Tumor and plasma cfDNA samples showed 70% concordance for mutation detection. Fourteen (39%) out of 46 samples showed a discordance for mutation detection between plasma cfDNA and tumor tissue. A total of 43 mutations were detected in the tissue biopsy specimen, but not in plasma cfDNA. Time difference between tissue biopsy specimen and plasma collection for these discordance cases range from 14 to 1330 days In order to improve specificity and avoid false positives, we strictly set the cutoff to 1% allelic frequency, 250X coverage and 25X variant coverage for NGS to avoid false positives. These cut off for cfDNA genotyping using NGS were set on the basis of limit of detection studies carried out using a serially diluted positive cfDNA samples [29]. 6 mutations were observed in cfDNA but were absent in the tissue specimen: *TP53p.Y220C* (1.5%) *EGFRp.E602G* (5%), *EGFRp.F712S* (1%), *PIK3CAp.E542K* (1.1%), *TP53p.R273H* (1%) and *APCp.*A1582P (5%). These mutations were not detected in the tissue specimen by manual observation performed in IGV. However 5 out of 6 cfDNA samples which showed additional clone are positive for other expected mutations. Time difference between tissue biopsy specimen and plasma collection for these discordance cases range from 1 −127 days. All *KRAS, BRAF, PIK3CA, EGFR, IDH1, IDH2, NRAS* and *AKT* mutations were validated with droplet ddPCR and UltraSEEK MassARRAY that provide higher sensitivity and lower cost than NGS [22, 30, 31]. NGS can interrogate many targets within a single reaction, but sensitive detection of minor variants at or below 1% frequency is difficult because of polymerase errors, and hence NGS requires a stringent bioinformatics pipeline and use of molecular barcodes to eliminate false positives. TAm-Seq, and CAAP Seq based NGS approaches showed a sensitivity of 2% to 0.5% [16, 17]. High-throughput platforms with higher analytical sensitivity, such as ddPCR, are capable of uncovering these minor variants and may reduce the false negative rate, but are limited in the number of targets that can be interrogated simultaneously, especially ddPCR. The UltraSEEK Oncogene panel can detect somatic mutations at clinically relevant levels with a performance that is equivalent to ddPCR, without compromising analytical sensitivity and accuracy using the same amount of input DNA [30, 32]. ddPCR showed higher analytical sensitivity as compared to MassARRAY and NGS and able to detect 6 variant calls which were detected as negative by NGS. ddPCR and MassARRAY showed 83% and 77% concordance with NGS for mutation detection with 100% and 79% sensitivity, respectively. MassARRAY showed 80% concordance for mutation detection with ddPCR. MassARRAY confirmed 4 out of 6 new variant calls detected by ddPCR. However, MassARRAY missed 4 variants detected by NGS and ddPCR. 3 out of 4 missed *PIK3CA* variants and hence reflect lower sensitivity of assay for *PIK3CA* variants.

We observed that cfDNA yield varied among different tumor types [9]. Many studies have shown that changes in cfDNA concentration can be correlated with development, prognosis, and survival of cancer patients. An increase of cfDNA concentration can be observed in patients with breast, gastric, lung, colon, and prostate cancers [21, 24, 33–35]. In this cohort, there was a marked correlation between cfDNA concentration and overall survival [36]. We observed a median OS for patients with lower cfDNA yield (< 30 ng) to be longer (74 vs 50 months; *P* = 0.03). Similarly, absence of mutations in plasma cfDNA was significantly longer than in patients with higher cfDNA yield and positive for mutation in cfDNA (74 vs 53 months; *P* = 0.04). A similar association between survival and ctDNA concentration has been reported in patients with advanced breast and metastatic colon cancers [9, 21]. Most (70%) patients in the cohort had at least one detectable mutation in plasma, which is consistent with earlier reports. cfDNA showed a similar mutation pattern to matched tissue biopsy specimen derived DNA.

In summary, this study demonstrates that targeted Ion Proton based ultra-deep sequencing of cfDNA and confirmation of variants by digital PCR enables more sensitive detection and monitoring of specific mutations in plasma cfDNA. We observed a good concordance among the different platform tested for mutation detection in plasma cfDNA. A strong correlation for mutation detection and allelic fraction was observed for tested methods. We have established sensitive detection of <1% minor allele frequency using ddPCR or UltraSEEK platforms with medium to high throughput capabilities. Clinical relevance of detection of variants < 1% in cfDNA in different solid tumors is yet to be explored. However, detection of low lying mutations in cfDNA can help in real time monitoring of response to therapy and evolution of new clones. With the availability of genomic profiles of patient tumors, use of ddPCR or MassARRAY in conjunction with NGS technology is an ideal approach for the rapid assessment and confirmation of mutations in plasma and can be applied in personalized medicine for predicting the prognosis and monitoring treatment efficacy of cancer patients.

## MATERIALS AND METHODS

### Study cohort

Peripheral blood was collected from 46 patients with diverse advanced cancers and known tumor mutation status. One hundred four tumor mutations were identified in using DNA from fixed, paraffin-embedded tumor tissue sections by using the semiconductor-based Ion PGM NGS platform with Ampliseq Cancer Hot Spot Panel v2 in a clinical molecular diagnostic laboratory. The tumors assessed were: 30 (65%) carcinomas, 7 (15%) melanomas, 5 (11%) sarcomas and 4 (9%) brain tumors. The carcinomas included adenocarcinomas of the colon (*n* = 14), breast (*n* = 8), pancreas (*n* = 2), appendiceal mucinous adenocarcinoma (*n* = 1), esophageal signet ring carcinoma (*n* = 1). Also included were 3 cases of squamous cell carcinoma of the tongue and 1 case of head and neck mucoepidermoid carcinoma. This study protocol was approved by the Institutional Review Board of MD Anderson Cancer Center and is consistent with international ethical standards on human subject's research. Informed consent was obtained from each study participant.

### Sample collection, cfDNA extraction and quantitation

10 ml peripheral blood was drawn from each patient in regular K3-EDTA tubes (BD Vacutainer, Becton Dickinson, NJ). Plasma was separated from blood within 16 hours. The plasma layer was carefully removed without disturbing the buffy coat, transferred to a new vial, and subjected to centrifugation at 2000Xg for 10 minutes at room temperature to remove residual cells. Plasma cfDNA was extracted from a 3-mL plasma sample using the QIAamp Circulating Nucleic Acid Kit (Qiagen, Valencia, CA) according to the manufacturer's instructions followed by elution in 50 μl. DNA was quantified by using the Qubit dsDNA HS Assay (Life Technologies, Illkirch, France).

### Ultra deep sequencing of cfDNA

10ng cfDNA was used to prepare libraries using the Ion Torrent Ampliseq 2.0 kit (ThermoFisher Scientific, Waltham, MA) and Ampliseq Cancer Hot Spot Panel v2 (CHPv2) according to the manufacturer's instructions. Samples were barcoded and quantified by qPCR using the Ion Xpress Barcode Adapter 1–96 kit and the Ion Library Taqman quantitation kit (ThermoFisher Scientific), respectively. Quantified libraries were pooled followed by e-PCR and sequenced on the Ion Proton using the Ion HI-Q PI Chip v3 and Ion PI HI-Q sequencing 200 kit (ThermoFisher Scientific) as described earlier [29].

Data analysis for NGS was performed as described earlier [29]. A cutoff of 300,000 reads with a quality score of AQ20 (one misaligned base per 100 bases) and a minimum sequencing depth of 250X was used as a measure of successful sequencing of a sample Sequencing results, mutations, and their respective allelic frequencies observed in cfDNA were compared with those identified in tumor tissue biopsy specimens to establish concordance.

### UltraSEEK MassARRAY

PCR was performed using 10 ng cfDNA per plex according to manufacturer's instructions (Agena Bioscience, San Diego, CA). Reactions were incubated initially at 94^°^C for 4 min. Forty-five cycles of PCR were performed at 94^°^C for 30 s, 56^°^C for 30 s, and 72^°^C for 1min. The PCR was completed with a final incubation of 5 minutes at 72^°^C. Thermocyling and incubation were performed in a GeneAmp PCR System 9700 (Thermo Fisher Scientific). Amplified products (5mL) were treated with shrimp alkaline phosphatase for 40 minutes at 37^°^C, followed by denaturation for 10 minutes at 85^°^C. Single-base extension was performed at 94^°^C for 30 s, followed by 40 cycles at 94^°^C for 5 s with five nested cycles of 52^°^C for 5 s, then 80^°^C for 5 s and incubation at 72^°^C for 3 min. Streptavidin-coated magnetic beads were used to capture the amplicon. Beads with captured products were pelleted using a magnet and, suspended with 13mL of elution solution, and incubated at 95^°^C for 5 minutes. Eluted products were conditioned with 5 mL (3 mg) of anion exchange resin slurry. Finally, the analyte was dispensed onto a Spectro CHIPArray solid support using a MassARRAY RS1000 Nano-dispenser. Data were acquired via matrix-assisted laser desorption/ionization time-of-flightmass spectrometry using the MassARRAY Analyzer. Data analysis were performed using Typer software version 4.0.26.74 (Agena Bioscience)

### Droplet digital PCR (ddPCR)

Genotyping was performed using 10 ng cfDNA on QX100 Droplet Digital PCR System (Bio-Rad, Hercules, CA). The primers and probes for detection of *KRAS, BRAF, PIK3CA, EGFR, IDH1, IDH2, NRAS* and *AKT* were obtained from Bio-Rad. PCR components were separated into individual reaction vessels using the QX100 Droplet Generator (Bio-Rad). The droplet generation process combines 70 μL of droplet generation oil with 22 μL of the ddPCR. 40 μl of formed droplet reaction was subjected to amplification. The cycling conditions for the PCR reaction included an initial incubation at 95°C for 10 minutes (min), 40 cycles of 94°C for 30 seconds (s) and 55°C for 60 s, and enzyme inactivation at 98°C for 10 min. After thermal cycling, the plates were transferred to a Droplet reader (Bio-Rad). The digital PCR data were analyzed with the Quanta Soft analytical software package (Bio-Rad).

### Limit-of-detection (LOD) by NGS, UltraSEEK and ddPCR

The LOD for the NGS platform has been performed using serially diluted samples as described earlier [29]. ddPCR and Ultrseek MassARRAY LOD study for mutation detection was performed using a serially diluted patient cfDNA sample positive for *KRAS G12D* mutation. The DNA was diluted with a cfDNA sample negative for these mutations to obtain samples with 50%, 25%, 12.5%, 6.25%, 3.15%, 1.5%, or 0.75% percent positive (mutated) DNA in wild-type DNA.

### Statistical and concordance analysis of tumor DNA and cfDNA

A paired *t*-test and Wilcoxon rank sum test were performed for statistical analysis to compare results among data sets. All analyses were performed with Graph Pad Prism software. Survival curves were calculated using the Kaplan–Meier method. The log-rank test was used to compare the survival curves. For statistical analysis of correlation between a pair of selected data for mutant allelic frequency in cfDNA was performed using Pearson correlation coefficient and *p* < 0.05 calculated by two tailed test were considered as significant.

## SUPPLEMENTARY MATERIALS TABLES








