# Supplementary material for: Detection of somatic mutations in cell-free DNA in plasma and correlation with overall survival in patients with solid tumors
Source: Oncotarget. 2017 Oct 24;9(12):10259–71. doi: 10.18632/oncotarget.21982 (PMC5828199; doi:10.18632/oncotarget.21982)
Supplement: Supplementary file 1 [file oncotarget-09-10259-s001.pdf]

Detection of somatic mutations in cell-free DNA in plasma and correlation with overall survival in patients with solid tumors

SUPPLEMENTARY MATERIALS

Supplementary Table 1: Clinical characteristic of studied subset (*N* = 46). See Supplementary_Table_1

Supplementary Table 2: Limit of detection study for ddPCR and MassARRAY using a serially diluted plasma cfDNA sample positive for *KRASp.G12D* mutation

	ddPCR		MassARRAY	
**Serial Dilution (%)**	**Observed FA**	**Expected FA**	**Observed NI**	**Expected NI**
**100**	38.6		14.5	
**50**	22.6	19.3	11.7	7.25
**25**	12.7	9.65	10	3.625
**12.5**	5.1	4.82	7.9	1.81
**6.25**	4.7	2.41	3.4	0.9
**3.12**	1.5	1.2	2.9	0.45
**1.5**	0.7	0.6	2.3	0.225
**0.75**	0.37	0.3	1.5	0.112

FA: Fractional Abundance; NI: Normalized Intensity.

Supplementary Table 3: Comparison of variant calls in tissue and plasma cfDNA. See Supplementary_Table_3

Supplementary Table 4: Discordance between the tissue and plasma samples for mutations detected. See Supplementary_Table_4

Supplementary Table 5: Comparison of variant calls detected by NGS, ddPCR and MassARRAY in plasma cfDNA

Samples	Organ origin Primary	Tissue histology Primary	Gene	SNP	Tissue AF	NGS	Coverage	ddPCR	Mass ARRAY
1	Brain	Oligoastrocytoma	*IDH1*	p.R132H	39.91	0.21	5218	1.9	1.9
2	Brain	Astrocytoma	*IDH1*	p.R132H	26.14	0	6761	0	0
3	Brain	Astrocytoma	*IDH1*	p.R132H	41.4	0	3287	0	0
4	Breast	Invasive ductal carcinoma	*PIK3CA*	p.H1047R	15.91	8.3	4408	8.1	1
5	Breast	Breast	*PIK3CA*	p.E545K	19.17	1.8	4577	6	1.1
6	Breast	Invasive ductal carcinoma; HER2+	*PIK3CA*	p.H1047R	27.35	30.6	2658	32.9	19
7	Colon, rectum	Colon	*KRAS*	p.G12D	11.64	33.7	3107	38	14.5
8	appendix	Colon	*KRAS*	p.G12D	22.32	0	3447	0.15	0
9	Rectum	Colon	*KRAS*	p.G13D	28.7	4	2027	8.2	6.8
			PIK3CA	p.E542K	15.09	1	3008	4.7	2.5
10	cecum & right-colon	Colon	*PIK3CA*	p.H1047R	31.73	1.9	3173	0.21	0
11	Colon, Sigmoid	Colon	*KRAS*	p.G12D	24.27	0	4278	0	0
12	Colon Right	Colon	*KRAS*	p.G12D	20.47	0	2080	0	0
13	Colon, rectum	Colon	*KRAS*	p.G12D	4.8	0	1726	0.3	0.5
14	rectum	Colon	*KRAS*	p.G12D	43.09	2.45	2186	2.7	3.5
15	Sigmoid	Colon	*KRAS*	p.G12V	18.51	0	1731	2.2	1.3
16	Sigmoid	Colon	*IDH2*	p.R172K	1.1	0	1538	0	0
17	Sigmoid	Colon	*IDH1*	p.R132C	21.05	1.5	3939	0.9	0.8
			KRAS	p.G12V	26.15	0.21	2344	0.4	0.8
18	Colon, cecal	Mucinous & NE	*BRAF*	p.V600E	41.12	0	992	1.6	0.5
19	Appendix mucinous	Mucinous Adenocarcinoma	*KRAS*	p.G12A	16.02	0	1133	0	0
20	Pancreas	Pancreas	*KRAS*	p.G12V	45.97	3	3131	2.1	0.1
21	Pancreas	Pancreas	*KRAS*	p.G12V	28.46	0	3050	0.68	1.6
22	Skin, Right calf	Melanoma	*BRAF*	p.V600E	30.65	1.3	2225	3.3	2.2
23	Skin upper back	Melanoma	*BRAF*	p.V600E	3	0	1960	0	0
24	Skin; Left shoulder	Melanoma	*BRAF*	p.V600E	44.8	0	3248	0.53	0
25	Skin; Left shoulder	Melanoma	*BRAF*	p.V600M	36.97	1.6	3214	2.1	1.2
			BRAF	p.V600E	36.83	0	2707	0	0
			IDH1	p.R132C	40.72	0	8103	0	0
26	Skin Left-arm	Melanoma	*NRAS*	p.61R	41.62	1	4421	0.6	0
27	Skin Right neck	Melanoma	*NRAS*	p.Q61R	58.28	24.4	7925	30.3	15.5
28	Skin Left-Great toe	Melanoma	*NRAS*	p.Q61K	45.83	0	4574	0	0
29	Tongue Head & Neck	SCC	*PIK3CA*	p.E545K	20.76	2.3	3770	1.2	0
30	H & N Tongue	SCC	*PIK3CA*	E545K	31.77	1.5	3656	1.3	0.6
31	Parotid salivary gland	Mucoepidermoid	*PIK3CA*	p.H1047R	26.75	1.2	3582	0.8	0

SCC: Squamous cell carcinoma.
