# Supplementary material for: Detection of somatic mutations in cell-free DNA in plasma and correlation with overall survival in patients with solid tumors
Source: Oncotarget. 2017 Oct 24;9(12):10259–71. doi: 10.18632/oncotarget.21982 (PMC5828199; doi:10.18632/oncotarget.21982)
Supplement: Supplementary file 2 [file oncotarget-09-10259-s002.docx]

**Supplementary Table 1: Clinical characteristic of studied subset (N=46)**

| **Samples** | **Tissue histology Primary** | **Organ origin Primary** | **Age** | **Gender** | **Time lapse (days)** | **Status** | **Pri Vs Met** | **cfDNA yield (ng/ml)** | **OS (Months)** |
| --- | --- | --- | --- | --- | --- | --- | --- | --- | --- |
| 1 | Oligoastrocytoma | Brain | 38 | F | 3011 | A | P | 7 | 121 |
| 2 | Astrocytoma | Brain | 43 | F | 60 | A | P | 7 | 61 |
| 3 | Astrocytoma | Brain | 53 | F | 15 | D | P | 25 | 9 |
| 4 | Astrocytoma anaplastic | Brain | 30 | F | 29 | A | P | 4 | 20 |
| 5 | Invasive ductal carcinoma | Breast | 58 | F | 9 | D | M | 38 | 41 |
| 6 | Invasive ductal carcinoma, triple negative | Left Breast | 69 | F | 227 | A | P | 27 | 31 |
| 7 | Invasive ductal carcinoma | Right Breast | 56 | F | 504 | D | R | 69 | 41 |
| 8 | Adenocarcinoma | Breast | 54 | F | 2145 | A | P | 4 | 94 |
| 9 | Carcinoma | Breast | 62 | F | 1 | D | R | 45 | 21 |
| 10 | Ductal Carcinoma | Breast | 59 | F | 1317 | D | P | 63 | 53 |
| 11 | Invasive ductal carcinoma; HER2+ | Brest | 38 | F | 7 | D | M | 32 | 60 |
| 12 | Invasive Carcinoma | Breast | 53 | F | 9 | D | R | 764 | 12 |
| 13 | Adenocarcinoma | Colon Appendix | 32 | M | 683 | D | P | 97 | 25 |
| 14 | Adenocarcinoma | colon | 56 | M | 1786 | A | M | 28 | 74 |
| 15 | Adenocarcinoma | Colon appendix |  | M | 35 | A | P | 27 | 24 |
| 16 | Adenocarcinoma | Colon, rectum | 82 | M | 470 | A | P | 59 | 77 |
| 17 | Adenocarcinoma | Colon, cecum & Right-colon | 75 | M | 239 | D | P | 135 | 11 |
| 18 | Adenocarcinoma | Colon, Sigmoid | 71 | M | 154 | A | M | 44 | 47 |
| 19 | Adenocarcinoma | Colon Right | 43 | F | 65 | A | P | 15 | 9 |
| 20 | Adenocarcinoma | Colon, rectum | 27 | F | 744 | A | P | 17 | 46 |
| 21 | Adenocarcinoma | Colon, rectum | 52 | F | 77 | D | P | 40 | 16 |
| 22 | Adenocarcinoma | Colon, Sigmoid | 72 | M | 21 | D | M | 81 | 111 |
| 23 | Adenocarcinoma | Colon, Right sigmoid | 51 | F | 36 | A | M | 9 | 10 |
| 24 | Adenocarcinoma | Colon, Sigmoid | 71 | M | 1585 | A | M | 39 | 89 |
| 25 | Adenocarcinoma | Colon, Sigmoid | 25 | F | 354 | A | M | 17 | 38 |
| 26 | Adenocarcinoma | Colon, cecal | 62 | M | 228 | A | P | 32 | 8 |
| 27 | Adenocarcinoma | Appendix mucinous | 78 | M | 1184 | D | P | 104 | 50 |
| 28 | Adenocarcinoma | Pancreas | 68 | F | 378 | D | M | 100 | 18 |
| 29 | Adenocarcinoma | Pancreas | 64 | F | 233 | D | P | 103 | 10 |
| 30 | Melanoma | Skin, Right calf | 32 | M | 10 | A | M | 17 | 55 |
| 31 | Melanoma | Skin right upper back | 43 | M | 1330 | A | M | 21 | 64 |
| 32 | Melanoma | Skin; Left shoulder | 50 | M | 85 | D | M | 41 | 99 |
| 33 | Melanoma | Skin; Left shoulder | 57 | M | 324 | A | M | 41 | 139 |
| 34 | Melanoma | Skin L-arm | 65 | M | 127 | A | M | 71 | 76 |
| 35 | Melanoma | Skin r neck | 36 | M | 253 | D | M | 19 | 18 |
| 36 | Melanoma | Skin auricular | 59 | F | 289 | A | M | 63 | 42 |
| 37 | Esophagus | Esophagus | 77 | M | 113 | D | R | 34 | 4 |
| 38 | Squamous cell carcinoma | Tongue H&N | 63 | M | 651 | D | P | 21 | 32 |
| 39 | Squamous cell carcinoma | Skin auricular | 75 | F | 32 | D | R | 73 | 41 |
| 40 | Squamous cell carcinoma | H & N Tongue | 66 | F | 232 | D | R | 54 | 36 |
| 41 | Mucoepidermoid | Parotid salivary gland | 56 | F | 262 | D | P | 64 | 12 |
| 42 | Leiomiosarcoma | Vesical wall | 49 | F | 316 | A | M | 27 | 74 |
| 43 | Leiomiosarcoma | Abdominal wall | 41 | F | 7 | A | M | 267 | 55 |
| 44 | Osteosarcoma | Bone Femur | 53 | M | 184 | A | R | 84 | 64 |
| 45 | Carcinosarcoma | Ovary | 68 | F | 498 | A | R | 61 | 109 |
| 46 | Condrosarcoma | Chest wall | 34 | F | 63 | A | M | 28 | 24 |

F: Female, M: Male, A: Alive, D: Dead, P: Primary, M: Metastatic, R: Recurrence, Time lapse (Days) from tissue biopsy to plasma collection, OS (Months) Overall survival in months from last follow up
