# Supplementary material for: Detection of somatic mutations in cell-free DNA in plasma and correlation with overall survival in patients with solid tumors
Source: Oncotarget. 2017 Oct 24;9(12):10259–71. doi: 10.18632/oncotarget.21982 (PMC5828199; doi:10.18632/oncotarget.21982)
Supplement: Supplementary file 3 [file oncotarget-09-10259-s003.docx]

**Supplementary Table 3: Comparison of variant calls in tissue and plasma cfDNA**

| **Samples** | **Tissue histology**  **Primary** | **Organ origin**  **Primary** | **Time difference (days)** | **Tumor stage** | **Gene** | **SNP** | **Tissue AF** | **Cov** | **Plasma AF** | **Cov** |
| --- | --- | --- | --- | --- | --- | --- | --- | --- | --- | --- |
| 1 | Oligoastrocytoma | Brain | 3011 | II | IDH1 | p.R132H | 39.9 | 4505 | 0.2 | 4000 |
| 2 | Astrocytoma | Brain | 60 | IV | IDH1 | p.R132H | 26.1 | 3677 | 0.0 | 6761 |
| 3 | Astrocytoma | Brain | 15 | III | PTEN | p.G129R | 56.3 | 856 | 0.0 | 5202 |
| 4 | Astrocytoma | Brain | 29 | III | IDH1 | p.R132H | 41.4 | 2000 | 0.0 | 3287 |
|  |  |  |  |  | TP53 | p.A161D | 83.9 | 1986 | 0.0 | 2350 |
|  |  |  |  |  | TP53 | del p.I162del | 84.3 | 1989 | 0.0 | 2352 |
| 5 | Invasive ductal carcinoma | Breast | 9 | I | PIK3CA | p.H1047R | 15.9 | 1219 | 8.3 | 4408 |
| 6 | Invasive ductal carcinoma, triple negative | Left Breast | 227 | II | PIK3CA | p.H1047L | 16.0 | 1215 | 0.0 | 3706 |
| 7 | Invasive ductal carcinoma | Right Breast | 504 | II | PIK3CA | p.Q546K | 27.6 | 2786 | 27.2 | 1322 |
|  |  |  |  |  | TP53 | p.R181H | 1.0 | 1804 | 0.0 | 1972 |
|  |  |  |  |  | TP53 | p.H179D | 53.6 | 1796 | 45.8 | 2567 |
| 8 | Adenocarcinoma | Breast | 2145 | II | PIK3CA | p.E545K | 19.2 | 1033 | 1.8 | 4577 |
| 9 | Carcinoma | Breast | 1 | IV | TP53 | p.R175H | 53.3 | 1999 | 5.0 | 5625 |
|  |  |  |  |  | TP53 | p.Y220C | 0.0 | 2068 | 1.5 | 5642 |
| 10 | Ductal Carcinoma | Breast | 1317 | III | TP53 | p.V216M | 25.3 | 1626 | 7.7 | 3632 |
|  |  |  |  |  | FBXW7 | p.L583F | 51.9 | 1719 | 57.4 | 6066 |
| 11 | Invasive ductal carcinoma; HER2+ | Breast | 7 | IV | PIK3CA | p.H1047R | 27.4 | 1700 | 30.6 | 2658 |
|  |  |  |  |  | APC | p..E1451K | 5.0 | 1079 | 30.6 | 3369 |
|  |  |  |  |  | TP53 | p.R248W | 32.5 | 2240 | 0.0 | 6565 |
| 12 | Invasive Carcinoma | Breast | 9 | IV | TP53 | p.C238Y | 78.2 | 2000 | 72.0 | 8000 |
| 13 | Adenocarcinoma | Colon Appendix | 683 | IV | KRAS | p.G12D | 11.6 | 2368 | 33.7 | 3107 |
|  |  |  |  |  | SMAD4 | p.R361C | 15.7 | 2198 | 38.9 | 3037 |
| 14 | Adenocarcinoma | colon | 1786 | IV | TP53 | p.R290H | 1.1 | 884 | 0.0 | 1747 |
| 15 | Adenocarcinoma | Colon appendix | 35 | III | KRAS | p.G12D | 22.3 | 1846 | 0* | 3447 |
|  |  |  |  |  | APC | del p.E1450fs* | 4.3 | 1737 | 0.0 | 3991 |
|  |  |  |  |  | GNAS | p.R201C | 38.4 | 1036 | 0.0 | 1506 |
|  |  |  |  |  | EGFR | p.E602G | 0.0 | 105 | 5.0 | 1144 |
| 16 | Adenocarcinoma | Colon, rectum | 470 | III | KRAS | p.G13D | 28.7 | 1993 | 4.0 | 2027 |
|  |  |  |  |  | PIK3CA | p.E542K | 15.1 | 1537 | 1.0 | 3008 |
| 17 | Adenocarcinoma | Colon, cecum & right colon | 239 | IV | APC | p.R1450* | 56.2 | 1781 | 0.0 | 3447 |
|  |  |  |  |  | KRAS | p.A146T | 64.2 | 1029 | 2.2 | 1768 |
|  |  |  |  |  | PIK3CA | p.H1047R | 31.7 | 1040 | 1.9 | 3173 |
|  |  |  |  |  | FBXW7 | p.L577S | 33.3 | 2198 | 0.0 | 7285 |
|  |  |  |  |  | APC | p.R1450* | 35.6 | 1986 | 0.0 | 2350 |
|  |  |  |  |  | PTEN | dupA p.T319fs*6 | 65.6 | 1592 | 0.0 | 2135 |
|  |  |  |  |  | SMAD4 | p.I525V | 74.3 | 1038 | 46.0 | 3197 |
| 18 | Adenocarcinoma | Colon, Sigmoid | 154 | III | KRAS | p.G12D | 24.3 | 2184 | 0.0 | 4278 |
|  |  |  |  |  | TP53 | p.R248Q | 25.2 | 2150 | 0.0 | 9078 |
|  |  |  |  |  | APC | del p.Q1291fs*14 | 37.4 | 1213 | 0.0 | 2276 |
| 19 | Adenocarcinoma | Colon Right | 65 | IV | KRAS | p.G12D | 20.5 | 2184 | 0.0 | 2080 |
| 20 | Adenocarcinoma | Colon, rectum | 744 | IV | APC | p.R1114* | 10.6 | 3450 | 0.0 | 3338 |
|  |  |  |  |  | KRAS | p.G12D | 4.8 | 1918 | 0* | 1726 |
|  |  |  |  |  | TP53 | p.2A>G | 5.9 | 2666 | 0.0 | 4286 |
| 21 | Adenocarcinoma | Colon, rectum | 77 | IV | APC | p.R876* | 37.5 | 1999 | 1.7 | 2059 |
|  |  |  |  |  | KRAS | p.G12D | 43.1 | 5676 | 2.5 | 2186 |
|  |  |  |  |  | TP53 | p.C238F | 14.5 | 1994 | 3.5 | 2878 |
|  |  |  |  |  | FGFR1 | p.R248W | 11.4 | 6025 | 1.0 | 4224 |
|  |  |  |  |  | PIK3CA. | p.E542K | 0.0 | 1885 | 1.1 | 2000 |
| 22 | Adenocarcinoma | Colon, Sigmoid | 21 | II | APC | del p.E1309fs*4 | 21.9 | 1978 | 4.0 | 1222 |
|  |  |  |  |  | KRAS | p.G12V | 18.5 | 3252 | 0* | 1731 |
|  |  |  |  |  | TP53 | p.R248Q | 25.2 | 2150 | 1.0 | 9078 |
|  |  |  |  |  | EGFR | p.F712S | 0.0 | 2648 | 1.0 | 3321 |
| 23 | Adenocarcinoma | Colon, Right sigmoid | 36 | IV | TP53 | p.C242fs*5 | 60.8 | 1784 | 7.6 | 1988 |
| 24 | Adenocarcinoma | Colon, Sigmoid | 1585 | IV | APC | p.E1309* | 11.8 | 652 | 15.0 | 1470 |
|  |  |  |  |  | BRAF | p.G466E | 1.2 | 1454 | 0.0 | 1850 |
|  |  |  |  |  | PIK3CA | p.R88Q | 1.0 | 1732 | 0.0 | 1035 |
|  |  |  |  |  | TP53 | p.E285K | 1.8 | 326 | 0.0 | 1965 |
|  |  |  |  |  | TP53 | p.C275F | 22.8 | 320 | 11.1 | 1969 |
|  |  |  |  |  | IDH2 | p.R172K | 1.1 | 635 | 0.0 | 1538 |
| 25 | Adenocarcinoma | Colon, Sigmoid | 354 | IV | IDH1 | p.R132C | 21.1 | 2816 | 1.5 | 3939 |
|  |  |  |  |  | KRAS | p.G12V | 26.2 | 1145 | 0.2 | 2344 |
| 26 | Adenocarcinoma | Colon, cecal | 228 | IV | BRAF | p.V600E | 41.1 | 1109 | 0* | 992 |
| 27 | Adenocarcinoma | Appendix mucinous | 1184 | IV | CDNK2A | p.R80* | 10.8 | 1272 | 0.0 | 1522 |
|  |  |  |  |  | GNAS | p.R201C | 23.7 | 658 | 0.0 | 2000 |
|  |  |  |  |  | KRAS | p.G12A | 16.0 | 2353 | 0.0 | 1133 |
|  |  |  |  |  | KRAS | p.T20M | 16.0 | 2369 | 0.0 | 1138 |
| 28 | Adenocarcinoma | Pancreas | 378 | IV | CDKN2A | p..L63R | 37.9 | 1508 | 30.5 | 1500 |
|  |  |  |  |  | KRAS | p.G12V | 46.0 | 1738 | 3.0 | 3131 |
| 29 | Adenocarcinoma | Pancreas | 233 | IV | KRAS | p.G12V | 28.5 | 1785 | 0* | 3050 |
| 30 | Melanoma | Skin, Right calf | 10 | IV | BRAF | p.V600E | 30.7 | 943 | 1.3 | 2225 |
|  |  |  |  |  | TP53 | p.R342P | 60.4 | 1162 | 2.3 | 4563 |
| 31 | Melanoma | Skin right upper back | 1330 | IV | BRAF | p.V600E | 3.0 | 1398 | 0.0 | 1960 |
|  |  |  |  |  | PTEN | p.Q17* | 3.6 | 1932 | 0.0 | 6488 |
| 32 | Melanoma | Skin; Left shoulder | 85 | IV | BRAF | p.V600E | 44.8 | 1000 | 0* | 3248 |
| 33 | Melanoma | Skin; Left shoulder | 324 | IV | BRAF | p.V600M | 37.0 | 2770 | 1.6 | 3247 |
|  |  |  |  |  | BRAF | p.V600E | 36.8 | 2767 | 0.0 | 2707 |
|  |  |  |  |  | CDKN2A | p.P114L | 62.0 | 113 | 0.0 | 3358 |
|  |  |  |  |  | IDH1 | p.R132C | 40.7 | 6709 | 0.1 | 8103 |
|  |  |  |  |  | TP53 | p.F134L | 39.0 | 3409 | 2.5 | 6290 |
| 34 | Melanoma | Skin Left-arm | 127 | IV | NRAS | p.61R | 41.6 | 1999 | 1.0 | 4421 |
|  |  |  |  |  | NRAS | p.Q61P | 32.3 | 1999 | 1.3 | 4421 |
|  |  |  |  |  | CDKN2A | p.R80* | 77.3 | 967 | 2.0 | 2650 |
|  |  |  |  |  | TP53 | p.R273H | 0.0 | 1783 | 1.0 | 3000 |
|  |  |  |  |  | TP53 | p.M237V | 19.2 | 820 | 11.9 | 1983 |
| 35 | Melanoma | Skin right neck | 253 | IV | NRAS | p.Q61R | 58.3 | 4477 | 24.4 | 7925 |
|  |  |  |  |  | TP53 | p.R248Q | 37.2 | 1800 | 12.1 | 7041 |
|  |  |  |  |  | HNF1A | p.S210F | 58.5 | 1561 | 21.6 | 6920 |
|  |  |  |  |  | SMAD4 | p.R361H | 18.8 | 1174 | 2.7 | 2539 |
| 36 | Melanoma | Skin auricular | 289 | IV | NRAS | p.Q61K | 45.8 | 1582 | 0.0 | 4574 |
| 37 | Esophagus | Esophagus | 113 | IV | TP53 | p.Y220C | 61.9 | 3627 | 1.3 | 6106 |
| 38 | SCC | Tongue H&N | 651 | II | FBXW7 | p.R479P | 38.0 | 1988 | 1.6 | 9768 |
|  |  |  |  |  | PIK3CA | p.E545K | 20.8 | 1209 | 2.3 | 3770 |
| 39 | SCC | Skin auricular | 32 | II | CDKN2A | c.151-2A>G | 78.5 | 478 | 0.0 | 3865 |
|  |  |  |  |  | HRAS. | p.Q61K | 65.9 | 452 | 0.0 | 3908 |
|  |  |  |  |  | TP53 | p.R248W | 49.8 | 1731 | 0.0 | 7090 |
|  |  |  |  |  | PIK3CA | p.Q546H | 28.5 | 1262 | 0.0 | 3442 |
|  |  |  |  |  | TP53 | p..N235K | 17.0 | 1721 | 0.0 | 7100 |
|  |  |  |  |  | IDH2 | p.R149W | 7.2 | 609 | 0.0 | 3491 |
| 40 | SCC | H & N Tongue | 232 | II | PIK3CA | p.E545K | 31.8 | 617 | 1.5 | 3091 |
|  |  |  |  |  | JAK3 | p.V722I | 48.5 | 611 | 50.3 | 2982 |
| 41 | Mucoepidermoid | Parotid salivary gland | 262 | II | HRAS | p.G13R | 35.1 | 1451 | 1.5 | 5247 |
|  |  |  |  |  | PIK3CA | p.H1047R | 26.8 | 2082 | 1.2 | 3582 |
|  |  |  |  |  | PIK3CA | p.V344G | 29.3 | 2997 | 1.5 | 3576 |
|  |  |  |  |  | TP53 | p.R175H | 19.1 | 2000 | 3.8 | 6423 |
| 42 | Leiomiosarcoma | Vesical wall | 316 | III | RB1 | p.R579* | 70.8 | 938 | 1.0 | 3265 |
|  |  |  |  |  | APC | p.A1582P | 1.0 | 769 | 5.0 | 1601 |
| 43 | Leiomiosarcoma | Abdominal wall | 7 | II | APC | p.A1497A | 78.4 | 1998 | 0.0 | 5548 |
| 44 | Osteosarcoma | Bone Femur | 184 | II | TP53 | p.R249M | 34.6 | 1056 | 0.0 | 10620 |
| 45 | Carcinosarcoma | Ovary | 498 | II | TP53 | p.K132Q | 80.9 | 1926 | 1.2 | 3000 |
| 46 | Condrosarcoma | Chest wall | 63 | II | TP53 | p.L348S | 69.8 | 739 | 0.0 | 8240 |

SCC: Squamous cell Carcinoma; H& N Head and Neck, Time difference (days) is the time difference between tissue and plasma collection *Variant call negative by NGS but positive by ddPCR in Plasma cfDNA, Cov: Coverage
