# Supplementary material for: Detection of somatic mutations in cell-free DNA in plasma and correlation with overall survival in patients with solid tumors
Source: Oncotarget. 2017 Oct 24;9(12):10259–71. doi: 10.18632/oncotarget.21982 (PMC5828199; doi:10.18632/oncotarget.21982)
Supplement: Supplementary file 4 [file oncotarget-09-10259-s004.docx]

**Supplementary Table 4: Discordance between the tissue and plasma samples for mutations detected**

| **Discordant variant calls positive in tumor tissue DNA but negative in plasma cfDNA** | | | | | | | | | | | |
| --- | --- | --- | --- | --- | --- | --- | --- | --- | --- | --- | --- |
| **Sample** | **Tumor Type** | **Time lapse (days)** | **cfDNA yield (ng/ml)** | **Gene** | **SNP** | | **Tissue AF** | | **Cov** | **Plasma AF** | **Cov** |
| 2 | Brain | 59.5 | 7.0 | IDH1 | p.R132H | | 26.14 | | 3677 | 0 | 6761 |
| 3 | Brain | 14.7 | 24.9 | PTEN | p.G129R | | 56.31 | | 856 | 0 | 5202 |
| 4 | Brain | 29.4 | 4.1 | IDH1 | p.R132H | | 41.4 | | 2000 | 0 | 3287 |
|  |  |  |  | TP53 | p.A161D | | 83.89 | | 1986 | 0 | 2350 |
|  |  |  |  | TP53 | del p.I162del | | 84.26 | | 1989 | 0 | 2352 |
| 6 | Breast | 226.8 | 27.0 | PIK3CA | p.H1047L | | 15.97 | | 1215 | 0 | 3706 |
| 14 | Colon | 1786 | 28 | TP53 | p.R290H | | 1.1 | | 884 | 0.0 | 1747 |
| 18 | Colon | 154 | 44 | KRAS | p.G12D | | 24.3 | | 2184 | 0.0 | 4278 |
|  |  |  |  | TP53 | p.R248Q | | 25.2 | | 2150 | 0.1 | 9078 |
|  |  |  |  | APC | del p.Q1291fs*14 | | 37.4 | | 1213 | 0.0 | 2276 |
| 19 | Colon | 65.1 | 14.7 | KRAS | p.G12D | | 20.47 | | 2184 | 0 | 2080 |
| 27 | Mucinous adenocarcinoma | 1183.7 | 103.8 | CDNK2A | p.R80* | | 10.79 | | 1272 | 0 | 1522 |
|  |  |  |  | GNAS | p.R201C | | 23.66 | | 658 | 0 | 2000 |
|  |  |  |  | KRAS | p.G12A | | 16.02 | | 2353 | 0 | 1133 |
|  |  |  |  | KRAS | p.T20M | | 16 | | 2369 | 0 | 1138 |
| 31 | Melanoma | 1330 | 21 | BRAF | p.V600E | | 3.0 | | 1398 | 0.0 | 1960 |
|  |  |  |  | PTEN | p.Q17* | | 3.6 | | 1932 | 0.0 | 6488 |
| 36 | Squamous cell carcinoma | 289.1 | 62.7 | NRAS | p.Q61K | | 45.83 | | 1582 | 0 | 4574 |
| 39 | Squamous cell carcinoma | 32 | 73 | CDKN2A | c.151-2A>G | | 78.5 | | 478 | 0.1 | 3865 |
|  |  |  |  | HRAS. | p.Q61K | | 65.9 | | 452 | 0.3 | 3908 |
|  |  |  |  | TP53 | p.R248W | | 49.8 | | 1731 | 0.2 | 7090 |
|  |  |  |  | PIK3CA | p.Q546H | | 28.5 | | 1262 | 0.0 | 3442 |
|  |  |  |  | TP53 | p..N235K | | 17.0 | | 1721 | 0.1 | 7100 |
|  |  |  |  | IDH2 | p.R149W | | 7.2 | | 609 | 0.0 | 3491 |
| 43 | Leiomiosarcoma | 7 | 41 | APC | p.A1497A | | 78.4 | | 1998 | 0.0 | 5548 |
| 44 | Osteosarcoma | 184 | 53 | TP53 | p.R249M | | 34.6 | | 1056 | 0.0 | 10620 |
| 46 | Condrosarcoma | 63 | 28 | TP53 | p.L348S | | 69.8 | | 739 | 0.1 | 8240 |
| **Discordant variant calls positive in plasma cfDNA but negative in tumor tissue DNA** | | | | | | | | | | | |
| **Sample** | **Tumor Type** | **Time lapse**  **(days)** | **cfDNA yield (ng/ml)** | **Gene** | **SNP** | **Tissue AF** | | **Cov** | | **Plasma AF** | **Cov** |
| 9 | Breast | 1 | 45.3 | TP53 | p.R175H | 53.28 | | 1999 | | 5 |  |
|  |  |  |  | TP53 | p.Y220C | 0 | | 2068 | | 1.5 |  |
| 15 | Colon | 35 | 27.4 | KRAS | p.G12D | 22.32 | | 1846 | | 0* | 3447 |
|  |  |  |  | APC | del p.E1450fs* | 4.32 | | 1737 | | 0 | 3991 |
|  |  |  |  | GNAS | p.R201C | 38.42 | | 1036 | | 0 | 1506 |
|  |  |  |  | EGFR | p.E602G | 0 | | 105 | | 5 | 1144 |
| 21 | Colon, | 77 | 40.0 | APC | p.R876* | 37.52 | | 1999 | | 1.7 | 2059 |
|  |  |  |  | KRAS | p.G12D | 43.09 | | 5676 | | 2.45 | 2186 |
|  |  |  |  | TP53 | p.C238F | 14.49 | | 1994 | | 3.5 | 2878 |
|  |  |  |  | FGFR1 | p.R248W | 11.39 | | 6025 | | 1 | 4224 |
|  |  |  |  | PIK3CA. | p.E542K | 0 | | 1885 | | 1.1 | 2000 |
| 22 | Colon | 21 | 80.7 | APC | del p.E1309fs*4 | 21.94 | | 1978 | | 4 | 1222 |
|  |  |  |  | KRAS | p.G12V | 18.51 | | 3252 | | 0* | 1731 |
|  |  |  |  | TP53 | p.R248Q | 25.21 | | 2150 | | 1 | 9078 |
|  |  |  |  | EGFR | p.F712S | 0 | | 2648 | | 1 | 3321 |
| 34 | Melanoma | 126.7 | 71.2 | NRAS | p.61R | 41.62 | | 1999 | | 1 | 4421 |
|  |  |  |  | NRAS | p.Q61P | 32.32 | | 1999 | | 1.3 | 4421 |
|  |  |  |  | CDKN2A | p.R80* | 77.25 | | 967 | | 2 | 2650 |
|  |  |  |  | TP53 | p.R273H | 0 | | 1783 | | 1 |  |
| 43 | Leiomiosarcoma | 316 | 27.0 | APC | p.A1582P | 1.0 | | 769 | | 5.0 | 1601 |

*Variant call negative by NGS but positive by ddPCR in Plasma cfDNA, cov: coverage
